# Improving consumer stickiness in livestream e-commerce: A mixed-methods study

**DOI:** 10.3389/fpsyg.2022.962786

**Published:** 2022-08-30

**Authors:** Lihong Shen, Yuning Zhang, Ying Fan, Yiduo Chen, Yi Zhao

**Affiliations:** ^1^College of Science and Technology, Ningbo University, Ningbo, China; ^2^Ningbo City College of Vocational Technology, Ningbo, China

**Keywords:** stickiness, livestream e-commerce, mixed-methods study, expectation confirmation theory, SmartPLS, playfulness

## Abstract

With the continuous development and improvement of Internet media technologies in China, the influence of livestream e-commerce is becoming increasingly prominent, and an increasing number of people are engaging in consumption activities in this field. It is important to study consumer stickiness in livestream e-commerce to promote economic structure adjustment and innovation-driven development. Therefore, in this study, we adopted the expectation confirmation theory (ECT) as the theoretical framework and analyzed the ECT and stickiness. The study considered satisfaction as the previous influencing factor of user and consumer stickiness, replaced the continuance intention in the expectation confirmation model with consumer stickiness as the explanatory variable, introduced the variable of perceived playfulness as the value perception after user experience, and established a consumer stickiness factors model. A total of 262 valid questionnaires were collected in this study, and SmartPLS analysis along with interviews were used to justify the limitations of data analysis. The results of the study demonstrated a significant effect of perceived usefulness and confirmation on satisfaction, a significant effect of confirmation on perceived usefulness, a significant effect of satisfaction on stickiness, and a significant effect of confirmation on perceived playfulness. Based on findings from the data analysis and interviews, we further proposed rationalized recommendations, and aimed to provide some theoretical guidance for future research on live streaming.

## Introduction

Due to the COVID-19 outbreak in 2020, China started implementing the long-term home quarantine policy. During this period, the nationwide economic stagnation promoted the development of short videos and online streaming, making up for the need to gather offline at physical stores. Certain terms such as “livestream selling,” “streamers,” and “stay-at-home economy” occupied the Hot Search List on Weibo; additionally, there was an explosive growth in the livestream e-commerce industry, successfully transforming the traditional consumer industry. Xia (2021) stated that China’s livestream e-commerce industry has undergone the following three stages: the initial exploration period, acceleration period, and explosion period, followed by the current stage of the industry characterized by the overall trends of normalization, industrialization, and competition. As a basic operation of major Internet live streaming platforms, livestream e-commerce—with the advantage of being able to rapidly improve the conversion rate—has been skyrocketing, forming a new marketing method with unique characteristics and greatly fostering the development of the consumer goods industry. According to data from the China Internet Network Information Centre (CNNIC), as of March 2020, the number of Internet users in China reached 904 million, and there were 262 million users of livestream e-commerce, which emerged in 2019 and achieved rapid development, accounting for 29.3% of all Internet users, 37.2% of online shopping users, and 47.3% of livestream users. As a result of the COVID-19 outbreak in 2020, advertisers affected by the pandemic switched to livestreaming to sell their products, and government departments also began livestreaming to attract business and promote agricultural products; thus, livestream selling has rapidly become popular (China Internet Development Statistics Report, 2022). At this stage, the study of online streaming and other related businesses has become a focus of research in various sectors, and the implied economic benefits have attracted people to explore its advantages. The endlessly emerging live-streaming platforms and content continue to satisfy consumers’ demands; while existing research has focused on justifying the purchasing behavior in the livestream e-commerce environment through theories such as SOR, ELM, or TAM (Xu et al., 2020, 2021; Gao et al., 2021; Chen et al., 2022), no studies have focused on the factors contributing to stickiness and continuous purchase after viewing e-commerce livestreams. In previous research, the expectation confirmation theory (ECT) is one of the most common theories used to explore the purchasing behavior in e-commerce for predicting and interpreting satisfaction and continuous behavior (Lin et al., 2009). However, the continuous purchasing behavior no longer effectively justifies the meaning in the context of livestream e-commerce, especially as the selling behavior of many livestream e-commerce businesses targets building consumer stickiness, through which continuous viewing and purchasing behavior are generated. Therefore, based on the ECT and replacement of continuance intention in the expectation confirmation model with stickiness as the explanatory variable, this study introduced perceived playfulness into the perceived value after user experience in the ECT theory, thus forming a theoretical model; thereafter, we analyzed, and put forward feasible recommendations for the factors contributing to consumer stickiness in livestream e-commerce. In summary, the main aim of the present study was to explore the factors affecting consumer stickiness to livestream e-commerce based on the ECT, and we propose the following research questions:

(1) What are the factors affecting stickiness to livestream e-commerce based on the ECT?
(2) In livestream e-commerce, does perceived playfulness have an impact on consumer satisfaction?

## Research model and hypotheses

### Research model

**FIGURE 1:**
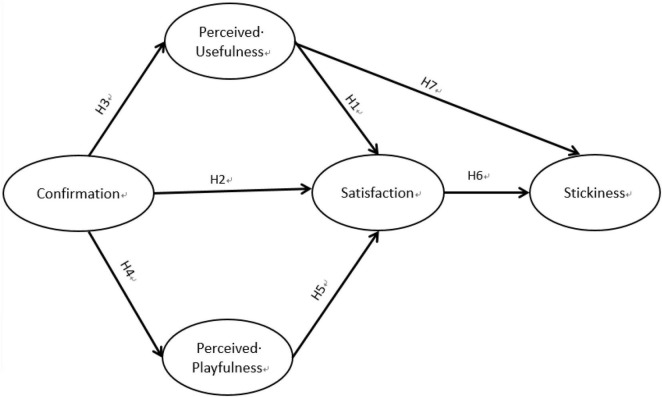
Research model.

In the present study, the research subjects were university students who have purchased from livestream e-commerce platforms. This study explored the stickiness of university students to livestream e-commerce through a combination of ECT (Bhattacherjee, 2001) and perceived playfulness (Moon and Kim, 2001) in the context of Internet entrepreneurship. The proposed study structure is shown in Figure 1. The operational definitions of the variables under the research model are described in Table 1.

**TABLE 1:** Operational definitions.

Variables	Operational definitions	Questionnaire items	Sources
Stickiness	Consumers repeatedly visit and perform consumption activities	1. I spend more time on livestreaming sites compared to other sites 2. I will visit as many livestreaming sites as possible 3. I intend to visit a livestreaming site every time I go online 4. I intend to extend my time spent on livestreaming sites	Yang and Lin, 2014
Perceived playfulness	Consumers feel entertained, focused, and curious during the live streaming	1. I do not realize the passage of time while watching livestreams 2. Watching livestreams provides me with pleasure 3. Some live streaming content is new to me	Lin et al., 2005
Satisfaction	Consumers’ subjective assessment of the quality of their live streaming experience	How do you feel about your overall experience of livestreaming? 1. Very happy/very unhappy 2. Very fulfilled/very unfulfilled 3. Very terrible/very pleasant 4. Very dissatisfied/very satisfied	Bhattacherjee, 2001
Perceived usefulness	The actual benefits perceived by users after using a certain technology or service	1. Through livestreaming, I can be better informed about information updates 2. Through livestreaming, I can decide more quickly and easily whether I want to use the information obtained 3. Through livestreaming, I can decide better than previously whether to use the information obtained 4. Through livestreaming, I can decide more quickly and easily than previously whether I want to use the information obtained 5. Through livestreaming, I can better decide whether to use the information obtained	Wang and Lin, 2012
Confirmation	The gap between what consumers expect and the actual service they receive during the real engagement process	1. My experience with livestreaming is better than I expected 2. The service provided by livestreaming is better than what I expected 3. Overall, most of my expectations for livestreaming have been fulfilled	Bhattacherjee, 2001

### Research hypotheses

Oliver (1980) stated that perceived usefulness is defined as the actual benefit that users perceive after using a certain technology or service, and that the higher the benefit for users, the greater their satisfaction. Bhattacherjee (2001) proposed that, in the process of applying ECT to the research on information system continuance intention, perceived usefulness replaces the original expectation, but is consistent with what was in the original expectation confirmation model because perceived usefulness essentially includes the expectation of the information system. A review of the relevant and existing literature on ECT indicated that perceived usefulness is significantly associated with both satisfaction and continuance intention (Bhattacherjee, 2001; Kim, 2010; Lee and Kwon, 2011; Mouakket, 2015; Wang et al., 2021). Based on these research findings, we concluded that perceived usefulness is an important factor affecting satisfaction and stickiness. Specifically, in livestream e-commerce, a consumer will feel satisfied if the actual benefits they receive while watching a livestream meet their needs. Based on this, the study proposed Hypotheses 1 and 7 (H1 and H7):

H1: Consumers’ perceived usefulness of livestream e-commerce has a positive impact on consumer satisfaction in livestream e-commerce.

H7: Consumers’ perceived usefulness of livestream e-commerce has a positive impact on consumer stickiness in livestream e-commerce.

Bhattacherjee (2001) believed that confirmation is the gap between participants’ perceived expectations and the actual services they receive during participation. In the context of the present study, this means that a consumer will have an expectation in mind before watching a livestream, and when they finish watching the livestream, the actual benefit they receive will be compared with their previous expectation, and confirmation means the difference between the two. Additionally, the results of previous studies confirmed that the degree of confirmation has a significant impact on perceived usefulness, and all these studies had good explanatory power (Lin and Wang, 2012; Lu et al., 2019). Hu and Zhang (2016) investigated Chinese university students’ continuance intention to use mobile reading apps, and demonstrated that confirmation affects the perceived usefulness and satisfaction. Once the expectation is confirmed, the consumer also perceives that the service has created a pleasurable experience for them; therefore, in livestream e-commerce, the consumer’s confirmation may influence their satisfaction, perceived usefulness, and perceived playfulness. This study also proposed the following hypotheses (H2 to H4):

H2: Confirmation has a positive impact on satisfaction in livestream e-commerce.

H3: Confirmation has a positive impact on perceived usefulness in livestream e-commerce.

H4: Confirmation has a positive impact on perceived playfulness in livestream e-commerce.

The concept of perceived playfulness was developed by Moon and Kim based on playfulness, which focuses more on describing the degree of satisfaction of intrinsic motivation in the interaction between users and information systems (Moon and Kim, 2001). In this study, perceived playfulness refers to the level of pleasure perceived by consumers while watching livestreams. Presently, perceived playfulness has been substantially studied in the research on continuous Internet usage behavior, and studies have confirmed that perceived playfulness is an important internal psychological factor influencing Internet use (Lin et al., 2005; Wang and Lin, 2012); moreover, another study found that perceived playfulness plays a positive role in influencing people’s satisfaction with web portals and social networking sites, among others (Lin et al., 2013). When consumers feel physically and mentally content while watching livestreams, this will have a positive impact on satisfaction. Therefore, this study proposed the following hypothesis (H5):

H5: Perceived playfulness has a positive impact on consumer satisfaction in livestream e-commerce.

According to the previous literature on ECT, satisfaction is a vital factor influencing users’ reuse intention (Oliver, 1980; Oliver and Bearden, 1985). Bhattacherjee (2001), in his research on information system continuance intention, reported that users’ continuance intention is mainly determined by their satisfaction after the actual use. Subsequent studies on ECT have also demonstrated the significant effect of satisfaction on continuance intention (Wang et al., 2021; Yang and Lin, 2016; Lu et al., 2019; Ashrafi et al., 2020). Similarly, we considered stickiness a deeper concept of continuance intention in this study, and hence there is a positive association between satisfaction and stickiness. Thus, this study proposed the following hypothesis (H6):

H6: Satisfaction has a positive impact on consumer stickiness in livestream e-commerce.

### Construct operationalization

The questionnaire items in the present study were mainly developed based on modified scales used by Bhattacherjee (2001), Lin et al. (2005), Wang and Lin (2012); Yang and Lin (2014), and Wang et al. (2022). The questionnaire mainly included six variables with four items on stickiness, three items on perceived playfulness, five items on perceived usefulness, four items on satisfaction, three items on interactivity, and three items on confirmation. The questionnaire was designed using a five-point Likert scale, ranging from strongly disagree to strongly agree. To ensure that the questionnaire was designed appropriately, the questionnaire items were translated from the original scales, and to ensure accurate translation of the items, they were first translated into Chinese by 3 professors specializing in e-commerce, and then translated into English by a translator trained in Chinese-English translation. As the questionnaire was administered in China, it was translated into Chinese and the questions were adjusted to the study context so that respondents could easily read and respond to the items. A professional translator also performed a backward translation to ensure the accuracy of the original translation. The scales and operational definitions of the questionnaire items are presented in Table 1.

### Data collection

In this study, data were collected mainly from university students. An online questionnaire was used because of its convenience, faster way of obtaining responses, low cost, and accessibility to respondents (Jin et al., 2021; Su et al., 2021). The questionnaire was administered through WJX to students at a technical university in Ningbo, China from February to March 2022. To ensure the quality of data collection, the questionnaire was distributed on the online group of each class by the researcher, and students in the groups were asked to complete the questionnaire. The snowball method was used for sample collection. Additionally, to improve the quality of the returned questionnaires, we also provided a compensation of 1–2 RMB to each student who properly completed the questionnaire. Among the returned questionnaires, 262 were valid. To ensure the questionnaire quality, the study referred to the practices in Lin et al.’s (2021a) study, which indicated that the questionnaire should take at least 3 min to complete, and questionnaires will be treated as invalid if items were responded with same or extreme answers. Finally, the questionnaires that passed the rigorous screening described above were used in the final data analysis. In addition, we incorporated the qualitative research method of Miles and Huberman (1994), which is more open, in-depth, and specific than quantitative research, and is more capable of capturing the entire picture and dealing with complex socio-cultural contexts. The questionnaire respondents were mainly students of Chinese universities. By means of convenience sampling, 30 respondents were selected for telephone and WeChat interviews to supplement the findings of this study.

In terms of gender, 115 male respondents and 147 female respondents completed the questionnaire, representing 43.89 and 56.11% of the sample, respectively. Among all respondents, 216 learned about livestream consumption behavior through the Internet, accounting for 38.38%; 126 through the television, accounting for 19.22%; 130 through newspapers and magazines, accounting for 20.78%; 110 through participating in livestream knowledge advertising campaigns, accounting for 18.95%; and 68 through other channels, accounting for 9.56%. The results indicated that numerous people learned about livestream e-commerce purchasing behavior through television, the Internet, and livestream e-commerce knowledge advertising campaigns. Among the livestreaming platforms, the most frequently visited were TikTok and Kuaishou, accounting for 33.57 and 33.56%, respectively, followed by Taobao, accounting for 22.88%, and other live streaming platforms, accounting for 11.32%. This result showed that most people are familiar with the two third-party live streaming e-commerce platforms—TikTok and Kuaishou—which account for almost the same percentage. Finally, in terms of payment tools, the most frequently used was Alipay, accounting for 64.38%, followed by WeChat, accounting for 32.97%, and others accounting for only 2.56%. This result demonstrated that most people use Alipay to make their purchases, followed by WeChat.

## Results

Partial least squares-SEM is a more relaxed use of CB-SEM for model estimation, which has met the assumption of a normal distribution, and has the ability to estimate more complex models using smaller samples (Hair et al., 2019; Shiau et al., 2019). Therefore, SmartPLS 3.3 software was used for the analysis of the structural and measurement models (Bido et al., 2014). In this study, the Harman’s single-factor test was used to test for common method variance using principal component analysis (Podsakoff et al., 2003). Based on the results, the factor explained below 40% of the variance, suggesting that there was no significant common method variance in this study (Shiau and Luo, 2012). In addition, in terms of detecting multicollinearity, this study referred to the recommendation of Hair et al. (2017), who suggested that the indicator should be under 5, and all variables in this study were in compliance with the suggested indicator.

### Measurement model

This model examines the relationship between latent and explicit variables, and therefore, confirmatory factor analysis (CFA) was used to explore the reliability of the questionnaire. CFA is a mainstream analytical approach that includes Factor Loading, Cronbach’s α, Composite Reliability (CR), and Average Variance Extracted (AVE). First, this study examined the factor loadings of the corresponding questionnaire items in SmartPLS. Normally, standardized factor loadings are higher, requiring the value to be greater than 0.7 (Hair et al., 2017). The results of the factor loadings in the present study are shown in Table 2, with all factor loadings greater than 0.7; therefore, each measured item exhibited good structural validity.

**TABLE 2:** Factors loading scale.

Variables	Items	Factors loading
Confirmation	CON1	0.928
	CON2	0.906
	CON3	0.900
Satisfaction	SAT1	0.909
	SAT2	0.898
	SAT3	0.902
	SAT4	0.912
Perceived playfulness	PLAY1	0.928
	PLAY2	0.906
	PLAY3	0.900
Stickiness	STICKINESS1	0.916
	STICKINESS2	0.942
	STICKINESS3	0.918
	STICKINESS4	0.892
Perceived usefulness	PU1	0.903
	PU2	0.911
	PU3	0.922
	PU4	0.888
	PU5	0.906

According to the reliability testing standards, the value of Cronbach’s α represents the reliability of the scale, with a higher Cronbach’s α value indicating a higher reliability of the scale. Cronbach’s α is the average value of the split-half reliability coefficients obtained from all possible item classification methods of a scale and is often used to measure reliability. Typically, the value of Cronbach’s α ranges between 0 and 1. An α value of less than 0.6 indicates insufficient internal consistency reliability; a value of 0.7–0.9 indicates that the scale is relatively reliable; and if the value is greater than 0.9, the scale is very reliable (Hair et al., 2017). CR is a measure of the reliability level of the composite variables through factor loadings, and generally speaking, a CR value of 0.7 or above indicates good reliability (Hair et al., 2017; Jin et al., 2021). AVE was also calculated by measuring the factor loadings, which represents the value of convergent validity between the sample value from statistical sampling and the expected value, and an ACE value of greater than 0.5 indicates good convergent validity of the latent variable (Lin et al., 2021a; Su et al., 2021), as shown in Table 3.

**TABLE 3:** Cronbach’s α, composite reliability and AVE.

Construct	Cronbach’s α	Composite reliability	AVE
Confirmation	0.913	0.945	0.851
Perceived usefulness	0.937	0.955	0.842
Perceived playfulness	0.898	0.936	0.831
Satisfaction	0.927	0.948	0.819
Stickiness	0.946	0.958	0.821

Second, the main purpose of discriminant validity in reliability testing was to examine the extent to which the measurement variables discriminate between different variables. The criterion for assessing discriminant validity is that the square root of AVE between variables is greater than the correlation coefficient between the different variables measured (Hair et al., 2017). As seen in Table 4, the square roots extracted of the average variance were all greater than the correlation coefficients between the variables, indicating that the results for each variable had discriminant validity.

**TABLE 4:** Fornell and Larcker.

	Confirmation	Perceived playfulness	Perceived usefulness	Satisfaction	Stickiness
Confirmation	**0.923**				
Perceived playfulness	0.747	**0.917**			
Perceived usefulness	0.644	0.725	**0.911**		
Satisfaction	0.569	0.643	0.762	**0.905**	
Stickiness	0.637	0.594	0.805	0.812	**0.906**

The bolded values are representing the “Construct”.

### Structural model

To examine the study hypotheses, the bootstrap method in SmartPLS was used to evaluate the PLS results with 5,000 times of resampling (Hair et al., 2017). Based on the results in the figure below, the overall *R*^2^ for the study results was 61.2%, while the *R*^2^ for satisfaction was 0.698, the *R*^2^ for perceived playfulness was 0.415 and the *R*^2^ for perceived usefulness was 0.649. The results showed that the research model proposed in this study had a high degree of explanatory power and provided valuable statistical results.

**FIGURE 2:**
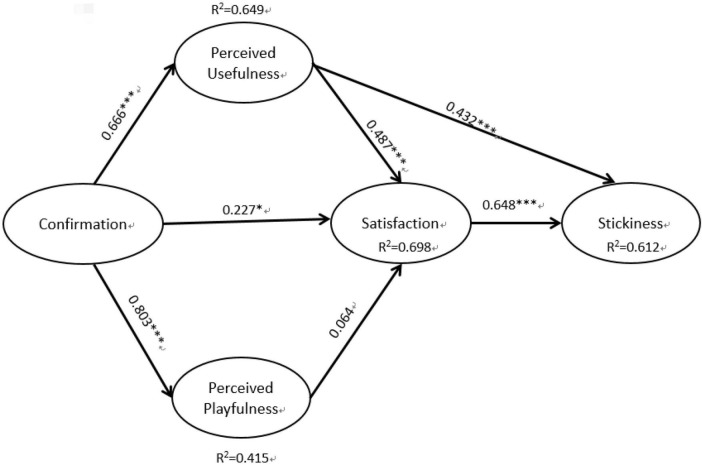
Partial least squares (PLS) results of the research model. *P < 0.05; ^**^P < 0.01; ^***^P < 0.001.

The structural model analysis is primarily used to explain the research hypotheses and to estimate the path coefficients between the variables. Structural models examine the relationships between variables (both latent and observed variables), also known as path analysis. In the process of path coefficient analysis, a p-value of the coefficient greater than 0.05 indicates that the effect between the two variables of the path is insignificant (Lin et al., 2021a,b). In this study, a partial least squares (PLS) regression analysis of the research data was conducted using SmartPLS, and considering the study model, a summary of the path coefficients was obtained as shown in Figure 2. In terms of hypothesis testing, perceived usefulness had a significant effect on satisfaction for H1 (β = 0.487, *p* < 0.001), confirmation also had a positive and significant effect on satisfaction for H2 (β = 0.227, *p* < 0.05), confirmation had a statistically significant effect on perceived usefulness for H3 (β = 0.666, *p* < 0.001), confirmation had a positive and significant effect on perceived playfulness for H4 (β = 0.803, *p* < 0.001), and perceived playfulness had no significant effect on satisfaction for H5 (β = 0.064 *p* > 0.05). Finally, satisfaction and perceived usefulness both had significant effects on stickiness for H6 (β = 0.446, *p* < 0.01) and H7 (β = 0.111, *p* > 0.05), respectively. Based on the above results, the extended model constructed in this study using expectation-confirmation theory as a basis for a framework with good explanatory power for understanding consumer stickiness in live-streaming e-commerce; the results of hypothesis testing are summarized in Figure 2 below.

## Qualitative research results

For the qualitative survey, this study randomly selected undergraduate students who returned the questionnaires as the interviewees for the follow-up results supplement. The interviews were conducted mainly *via* telephone and WeChat. Each interviewee was interviewed for 10–15 min about their experience in using live-streaming e-commerce. The results of this study showed that the interviewees generally felt that they were accustomed to using the live-streaming e-commerce platforms and most of them thought that the products sold on these e-commerce platforms were of better quality than those sold online alone. As interviewee A1 said, “For me, watching e-commerce live streaming has become a habit and it is easy to find high-quality products from live-streaming e-commerce”; interviewee A2 also said, “I started to watch e-commerce live streaming in my second year of college. Sometimes I find the introduction of the live streamers very interesting, and I am also easily attracted by the introduction of the live streamers and choose the products recommended by them.” Second, the interviewees who were senior undergraduate students also agreed that long-term attention to live-streaming e-commerce would develop stickiness; in particular, students studying business administration liked to study the business model of live streamers. For example, the senior student A18 said that watching live streaming has become a type of playfulness after work, through which she could also understand the current market situation and continuously observe the sales methods of the live streamers. The interviewee A22 also said that live streamers were very interesting when introducing products, and their introductions, in particular, inspired consumers’ purchase intention. The results of qualitative analysis showed that the hypothesis development of this study is accurate and most of the interviewees’ feedback is also consistent. Based on the results of the questionnaires and interviews, the results of this study are also discussed in the second half of this study.

## Research discussion and results

### Research discussion

Based on the empirical results, perceived usefulness had a significant effect on satisfaction (H1), which was consistent with the results of previous studies (Wang et al., 2021). The results of this study showed that in terms of the live-streaming e-commerce environment, the live-streaming e-commerce could provide valuable product information and services, which had an important influence on consumer satisfaction. Confirmation also had a positive and significant correlation with satisfaction (H2), which was consistent with the results of the previous study by Shiau et al. (2020). The results indicated that college students’ experience of using the live-streaming e-commerce services was better than they expected before using, so they were more satisfied with the content of the live-streaming e-commerce services. These results also indicated that when selecting products or operating policies, the live-streaming e-commerce platform should strengthen the selection of products that provide better experiences for college students, which will also generate a high level of recognition for the platform. Moreover, confirmation had a significant effect on perceived usefulness (H3), which was consistent with the findings of previous studies (Wang et al., 2022). For college students, watching the live streaming is based on positive feelings of the experience, which leads them to believe that the service is useful. Therefore, for live-streaming e-commerce platforms, more online interactive activities can be arranged to make consumers feel that they are recognized by the live streamers, which can also further increase the extent to which consumers are useful to live-streaming e-commerce.

According to the results of perceived playfulness, confirmation had a significant effect on perceived playfulness (H4), which was consistent with the previous findings of Lin et al. (2005) in studying the Internet environment. Therefore, this study revealed that college students perceived a better experience of using the live-streaming platforms, particularly insofar as the attractiveness of the live streamers also improved their pleasure in watching live streaming. However, perceived playfulness had no significant effect on satisfaction (H5), which was inconsistent with the findings of the study by Lin et al. (2005). The results indicated that although the playfulness of watching live streaming is an important factor, satisfaction still depends on the quality of the products sold by the live streamer, not just the playfulness value. Finally, satisfaction (H6) and perceived usefulness (H7) both had significant effects on stickiness, which was consistent with the findings of previous studies (Lu et al., 2019; Gupta et al., 2020). If college students perceive the content of the live streaming as useful and feel satisfied, they will generate stickiness to the live-streaming platform. Therefore, live streamers or live-streaming platforms may need to spend more effort on improving the quality and diversity of their products to sustain the stickiness of college students’ continued viewing.

### Implications for academic research

This study empirically investigated the influencing factors of consumer stickiness to live streaming from the perspective of live streamers. The content and data analysis of this study revealed that live-streaming platforms and live streamers need further improvement, therefore the following practical and theoretical suggestions are offered in view of the current live-streaming environment.

(1) Perceived usefulness and confirmation have the greatest impacts on consumer satisfaction and consumer stickiness can be improved by influencing the intermediate variable of satisfaction. Thus, live-streaming platforms and live streamers need to pay more attention to these two factors. That is, they should consider how to positively expand the gap between consumers’ expectations and the actual services received during the actual engagement and identify their own marketing strategies. It is necessary to ensure the quality of the goods or services so as to expand confirmation, namely, the actual benefits, to expand the gap between expectations and reality, and also to prevent excessive boasting regarding the goods or services, which raises consumers’ expectations.
(2) Playfulness may be a potential influencing factor. Playfulness does not have a direct effect on stickiness, but after college students confirm the live-streaming e-commerce platforms, the playfulness of the platforms will be generated. Therefore, enhancing the playfulness of the platform and the quality of products can help to obtain loyal consumers more accurately among college students and further generate consumer stickiness.
(3) Previous studies mainly used questionnaires to collect data and analyzed the data to explain the significance of the study. However, this study adopted a mixed methods approach, and from the college students’ interviews, we found that the usefulness discussed in the results was specifically related to the quality of products, especially as college students nowadays are careful with money. Therefore, from the perspective of college students, the content of each live-streaming product can be further refined, especially for products that young people prefer; this can increase customer stickiness and also stabilize the audience.

### Research limitation

The sample subjects and distribution of the questionnaires in this study were not broad enough. This study mainly distributed and collected questionnaires *via* WeChat and QQ, and the subjects were mainly the students in our university, who were relatively homogeneous. In future research, a more diverse sample database will be used. The sample size and the area of the survey should be expanded, and the diversity of the sample should be extended to improve the generalizability of the results. Second, this study mainly investigated how to improve consumer stickiness in the live-streaming model based on the expectation-confirmation theory. In the future, studies can build models based on multiple theories, focus on the internal relationships between independent variables, and improve the research methods to improve the generalizability of the results.

## Data availability statement

The original contributions presented in this study are included in the article/supplementary material, further inquiries can be directed to the corresponding authors.

## Author contributions

LS and YZ designed the research, provided guidance throughout the entire research process, participated in the literature collection, analysis, and organization. YNZ and YF collected the references, did the literature analysis, and wrote the manuscript. YC helped with translation and offered modification suggestions. All authors listed have made a substantial, direct, and intellectual contribution to the work, and approved it for publication.
